# Changes in Soil Bacteriobiome in Response to Organic Amendments and Cd^2+^ Stress

**DOI:** 10.3390/ijms27135783

**Published:** 2026-06-26

**Authors:** Agata Borowik, Jadwiga Wyszkowska, Magdalena Zaborowska, Jan Kucharski

**Affiliations:** Department of Soil Science and Microbiology, Faculty of Agriculture and Forestry, University of Warmia and Mazury in Olsztyn, 10-719 Olsztyn, Poland; agata.borowik@uwm.edu.pl (A.B.); m.zaborowska@uwm.edu.pl (M.Z.); jan.kucharski@uwm.edu.pl (J.K.)

**Keywords:** bacterial diversity, cadmium-contaminated soil, compost, fermented bark, humic acids, metabolic functions

## Abstract

Cadmium contamination of soils poses a global threat to food security and ecosystem stability. Soil bacteria play a key role in mitigating Cd-induced stress, and their adaptive capabilities can be modulated by the application of organic amendments such as compost, fermented bark, or preparations containing humic acid. This article presents the results of studies on soil bacterial communities using culture-dependent and next-generation sequencing approaches. Based on the obtained data, colony development indices and ecophysiological diversity indices were determined for organotrophic bacteria and actinobacteria. Alpha and beta diversity of bacteria were also assessed, common and unique genera occurring in the studied soils were identified, and the predicted metabolic functions of microorganisms were determined. It was found that cadmium reduced the abundance of organotrophic bacteria and actinobacteria by 54.5% and 12.9%, respectively, compared to the control, resulting in a shift in the bacterial community structure from r-strategists toward K-strategists. Humic acid increased the abundance of organotrophic bacteria and actinobacteria by 42.8% and 57.3%. Compost most effectively mitigated cadmium effects by stabilizing the colony development index and bacterial ecophysiological diversity. Cadmium strongly altered the soil bacterial microbiome, reducing the abundance of *Actinomycetota* while increasing that of *Pseudomonadota* and *Bacteroidota*. The application of organic amendments influenced the bacterial response to Cd^2+^-induced stress. Fermented bark was associated with an increased abundance of *Sphingomonas*, whereas compost was associated with an increased abundance of *Cellulosimicrobium*. Although none of the organic amendments affected the overall diversity index under these conditions, compost improved the evenness and ecological stability of the bacterial community. The dominance of aerobic chemoheterotrophs involved in the carbon cycle and the degradation of organic compounds was demonstrated. Compost most effectively supported biogeochemical processes.

## 1. Introduction

Soil contamination with heavy metals, particularly cadmium, currently poses one of the most serious global threats to the stability of agricultural ecosystems and food security [1,2,3]. The presence of cadmium in soil results from the combined influence of natural (geogenic) processes and intensive human activities. The main cause of the drastic increase in cadmium accumulation in soils is the long-term application of phosphate fertilizers [4,5]. Additional inputs originate from wastewater and sewage sludge [6], as well as atmospheric deposition associated with industrial emissions, particularly non-ferrous metal smelting of zinc and lead ores [7], and fossil fuel combustion [8,9,10]. Cadmium is highly mobile in the soil profile, has no known biological function, and exhibits a long environmental half-life. Consequently, its continuous input leads to persistent accumulation in the root zone. This creates chronic selective pressure on the soil microbiome and poses a direct threat to food safety [1,6].

The application of organic matter significantly alters the microbial structure of cadmium-contaminated soils by stimulating the proliferation of indigenous soil bacteria. Rich in humic and biogenic substances, these amendments not only decrease the bioavailability of Cd^2+^ by immobilizing it within organo-mineral complexes, but also provide an energy substrate that helps maintain the functional biodiversity of soil microorganisms [11,12,13]. According to Li et al. [14], Cd exerts multiple toxic effects on microbial cells, including inhibition of DNA repair mechanisms and direct interference with nucleic acid structure, which leads to mutations and impaired replication and transcription. Furthermore, Cd inactivates enzymes due to its high affinity for sulfhydryl (–SH) groups in cysteine residues. Binding to these groups induces conformational changes in proteins, resulting in loss of catalytic activity in key metabolic enzymes. Furthermore, Cd^2+^ can displace Zn^2+^ and Ca^2+^ ions due to chemical similarity, thereby blocking membrane transporters and disrupting the cell’s mineral balance [6,15], e.g., of zinc and calcium [3].

In the search for sustainable strategies to mitigate heavy metal toxicity, considerable attention has been given to the use of organic amendments. Low-molecular-weight organic acids released from organic matter, such as threonic and oxalic acids, may enhance cadmium mobility by promoting desorption of Cd fractions bound to organic matter and iron oxides. This process can facilitate cadmium leaching from soil. In contrast, high-molecular-weight organic acids, including humic and fulvic acids, exhibit a strong capacity to immobilize cadmium, significantly reducing its bioavailability in soil solution [14].

Additives selected in studies, such as compost, fermented tree bark, or preparations based on concentrated humic acids (e.g., HumiAgra), can therefore serve a multifaceted purpose. Fermented bark, rich in lignocellulosic structures and phenolic compounds (tannins), creates specific protective micro-niches and provides unique metal-binding ligands [16,17,18]. Compost provides stabilized organic matter, including labile carbon, and a diverse, functionally beneficial bacterial community that stimulates rapid restoration of the microbiome structure [19,20,21]. Both fermented bark and compost increase the pool of humic and fulvic acids in the soil [22,23]. Highly reactive humic and fulvic acids can act as powerful natural chelators and significantly reduce the bioavailability of Cd^2+^ ions while simultaneously stimulating respiratory activity and cell division in stress-resistant bacteria [24,25]. According to Haroun et al. [22], they can also act as a molecular buffer; their numerous functional groups (carboxyl and hydroxyl) form stable, non-toxic complexes with cadmium, thereby drastically reducing the selective pressure on Cd-sensitive bacteria. It is also worth noting that the method used to obtain humic acids determines their chemical composition and functionality. According to Anielak et al. [26] acids derived from lignosulfonates are characterized by high oxygen content and excellent solubility, facilitating their application. Among coal types, lignite has the highest carbon content (approx. 70%), making it ideal for improving soil structure; however, its gel-like form makes it difficult to apply with precision in large-scale agriculture. The use of humic acids from sewage sludge fits within a circular economy, but such substances may contain micropollutants such as pharmaceuticals, polycyclic aromatic hydrocarbons (PAHs), and hormones, which require additional toxicity testing prior to their widespread implementation.

The use of organic additives may therefore promote less frequently described bacterial taxa, which, in response to the composition of fermented bark or humus preparations, will exhibit different adaptive strategies toward Cd^2+^ stress. Thus, despite numerous studies confirming the effectiveness of individual organic additives in phytoremediation and bioremediation, the processes occurring within the microbiome under Cd^2+^ stress remain incompletely understood. Therefore, our research combines traditional bacterial isolation methods, advanced sequencing techniques, and bioinformatics tools that rely on database searches and sequence homology algorithms to assess biodiversity, with a focus on detecting subtle changes in the microbiome among key bacterial taxa.

Previous studies have primarily focused on the effects of Cd^2+^ on plants and the overall structure of the microbiome, as well as on changes in soil physicochemical properties following the application of organic amendments. However, studies integrating taxonomic and functional approaches to identify specific taxa that respond to cadmium and various organic amendments remain scarce. To address this gap, the present study investigates whether compost, fermented bark, and humic acids applied as the HumiAgra preparation induce distinct microbial adaptive strategies. The study also aimed to link changes in the bacterial microbiome with detoxification processes and resistance to Cd contamination. The following research hypotheses were formulated: (1) cadmium contamination significantly affects the abundance, structure, diversity, and functional activity of the bacterial microbiome; (2) the application of compost, fermented bark, and humic acids in the form of the HumiAgra preparation modifies the response of bacterial communities to Cd-induced stress; (3) exogenous organic matter reduces the negative impact of Cd on soil bacteria, stabilizing their populations and supporting key biogeochemical processes; (4) the effectiveness of Cd stress mitigation depends on the type of organic matter applied.

The aim of this study was to comprehensively assess changes in the structure and functions of the soil microbiome under the influence of Cd^2+^ stress and organic amendments (compost, fermented bark, and HumiAgra), using classical bacterial isolation methods, next-generation sequencing, and functional analysis based on sequence homology algorithms and protein prediction (MACADAM). An additional objective is to identify adaptive responses among key bacterial taxa and evaluate whether different organic amendments promote distinct Cd^2+^ tolerance strategies.

## 2. Results

### 2.1. The Role of Organic Amendments in Shaping the Resistance of Cultured Bacteria to Excess Cadmium in Soil

**Figure 1:**
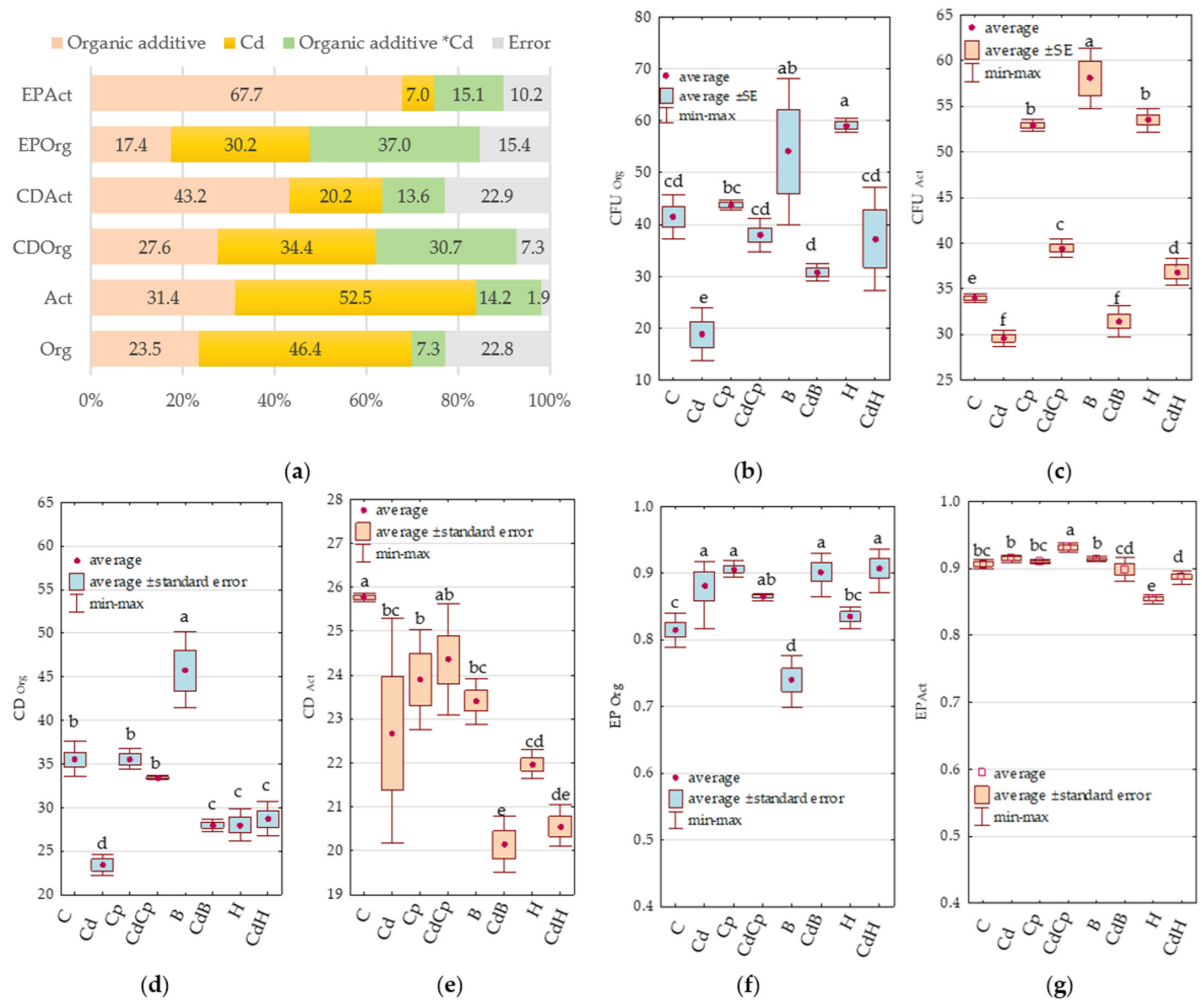
The effect of cadmium (Cd^2+^) and organic amendments on the abundance and diversity of organotrophic bacteria (Org) and actinobacteria (Act), measured using η^2^, in % (**a**), and the abundance of Org (**b**) and Act (**c**) expressed in 10^8^ CFU kg^−1^ d.m. of soil; colony development index (CD) of organotrophic bacteria (**d**) and actinobacteria (**e**) in the soil; ecophysiological diversity index (EP) of organotrophic bacteria (**f**) and actinobacteria (**g**) in the soil. C—uncontaminated soil, Cp—uncontaminated soil with compost, B—uncontaminated soil with fermented bark, H—uncontaminated soil with HumiAgra, Cd—soil contaminated with Cd^2+^, CdCp—soil contaminated with Cd^2+^ and compost, CdB—soil contaminated with Cd^2+^ and fermented bark, CdH—soil contaminated with Cd^2+^ and HumiAgra. Homogeneous groups denoted with letters (a–f) were calculated separately for each microbial group.

Determination of bacterial counts on microbiological media revealed the highest abundance of organotrophic bacteria in the soil following the application of the HumiAgra preparation (H) (Figure 1a). The application of H significantly increased (by 42.8%) the number of organotrophic bacteria CFUs compared to the control (C). High numbers of these bacteria were also observed after the application of fermented bark (B)—an increase of 30.5%, and compost (Cp)—an increase of 5.6%. In the case of actinobacteria, the greatest increase in their abundance was observed in the soil after the application of B (an increase of 70.8%), H (an increase of 57.3%), and Cp (an increase of 55.6%) relative to C (Figure 1b,c).

The introduction of Cd^2+^ into the soil resulted in a 54.5% decrease in the abundance of organotrophic bacteria and a 12.9% decrease in the abundance of actinobacteria compared with the control (C). In the presence of Cd^2+^, the addition of H significantly increased the abundance of organotrophic bacteria by 97.8% and actinobacteria by 24.6%, although these values remained lower than in H without Cd^2+^ (decreases of 36.9% and 31.0%, respectively). The addition of B to Cd^2+^-contaminated soil increased the abundance of organotrophic bacteria by 63.3% and actinobacteria by 6.3% compared with Cd^2+^, whereas in B without Cd^2+^, their abundance decreased by 43.1% and 45.8%, respectively. In CdCp, the abundance of organotrophic bacteria was 101.4% higher and that of actinobacteria 33.4% higher compared with Cd^2+^, but still lower than in Cp, where decreases of 13.2% and 25.4% were observed, respectively. These results are summarized by the η^2^ coefficient, which indicates the proportion of variance explained by each factor in ANOVA. The results show that cadmium had a stronger effect on cultivable bacteria than organic amendments, explaining 46.4% of the variation in organotrophic bacteria and 52.5% in actinobacteria. Organic amendments accounted for 23.5% and 31.4% of the variation, respectively. The CD index values also indicate significant changes in the growth strategies of microorganisms in response to the tested factors (Figure 1d,e). The colony development index (CD) for organotrophic bacteria was explained by Cd^2+^ (34.4%) and by organic supplements (27.6%) (Figure 1a). The CD index for actinobacteria was dependent on Cd^2+^ by 20.2% and on organic matter by 43.2%. Cd^2+^ altered microbial development strategies. The application of B increased CD values of organotrophic bacteria, whereas Cd^2+^ reduced CD values in both microbial groups. The smallest changes in CD values for both organotrophic bacteria and actinobacteria were observed in CdCp soil. Changes in the ecophysiological diversity (EP) index of organotrophic bacteria and actinobacteria are shown in Figure 1e,f. The lowest ecophysiological diversity (Org) was observed in soil amended with B, followed by C and H, whereas the highest values were recorded in the remaining treatments. In contrast to actinobacteria, the EP index of organotrophic bacteria increased under Cd^2+^ contamination. Cp and H also increased EP (Org), while B decreased it. Conversely, actinobacteria showed increased EP under B, whereas H significantly reduced it and Cp had no effect. The EP index of organotrophic bacteria was mainly explained by Cd^2+^ contamination (30.2%), whereas that of actinobacteria was mainly explained by organic amendments (67.7%).

### 2.2. The Role of Organic Amendments in Shaping the Resistance of Uncultivable Bacteria to Cadmium Excess in Soil

**Figure 2:**
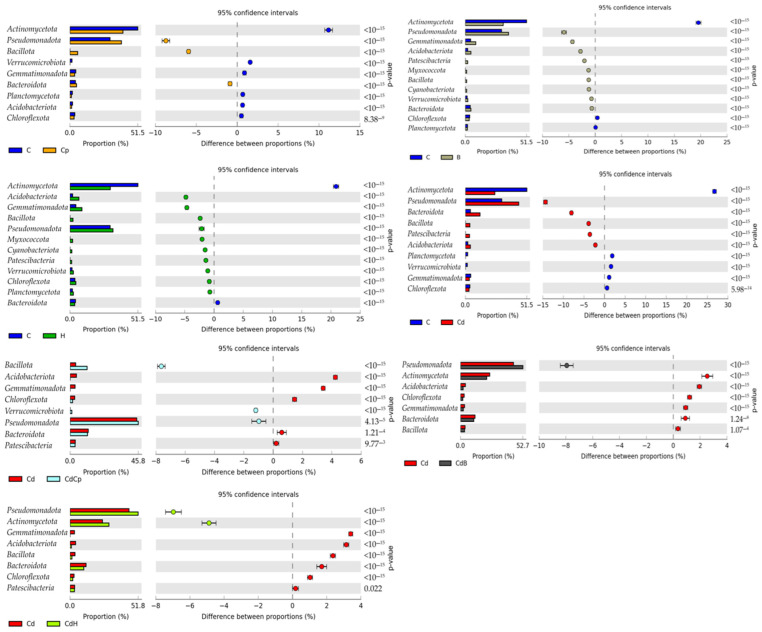
Effect of organic amendments and cadmium on the abundance of dominant bacterial types in soil. All presented data are statistically significant. C—uncontaminated soil, Cp—uncontaminated soil with compost, B—uncontaminated soil with fermented bark, H—uncontaminated soil with HumiAgra, Cd—soil contaminated with Cd^2+^, CdCp—soil contaminated with Cd^2+^ and compost, CdB—soil contaminated with Cd^2+^ and fermented bark, CdH—soil contaminated with Cd^2+^ and HumiAgra.

Analysis of bacterial communities at the phylum level revealed significant differences among the soil samples analyzed (Figure 2). A total of 30 bacterial phyla were identified, of which 12 constituted the dominant bacterial microbiome. *Actinomycetota*, *Pseudomonadota*, and *Bacteroidota* were present in all soil samples. It was observed that *Actinomycetota* in the uncontaminated control soil without organic amendments (C) accounted for 51.48%, whereas in soils amended with Cp, B, and H, their relative abundances decreased to 40.31%, 31.90%, and 30.62%, respectively. In Cd^2+^-contaminated soils, this abundance further decreased, reaching 29.62% in CdH, 24.86% in CdCp, 24.74% in Cd, and 22.20% in CdB. In contrast, *Pseudomonadota* and *Bacteroidota* showed an opposite trend, reaching their highest abundances in Cd^2+^-contaminated treatments. Analysis of *Actinomycetota* abundance after Cp application relative to the control revealed a decrease by 11.17%, whereas H and B reduced it by 20.87% and 19.58%, respectively. In contrast, *Pseudomonadota* increased by 8.71% in Cp, 5.92% in B, and 2.11% in H. Cd^2+^, compared with C, reduced the abundance of *Actinomycetota* by 26.75% and increased that of *Pseudomonadota* and *Bacteroidota* by 14.34% and 8.02%, respectively. The greatest effect on the increase in *Pseudomonadota* abundance in contaminated soils was observed after the application of B, where their abundance in CdB soil increased by 7.94% compared with Cd^2+^. Compost increased the abundance of *Bacteroidota* by 7.63%, while H increased the abundance of *Pseudomonadota* by 6.96% and *Actinomycetota* by 4.88%.

**Figure 3:**
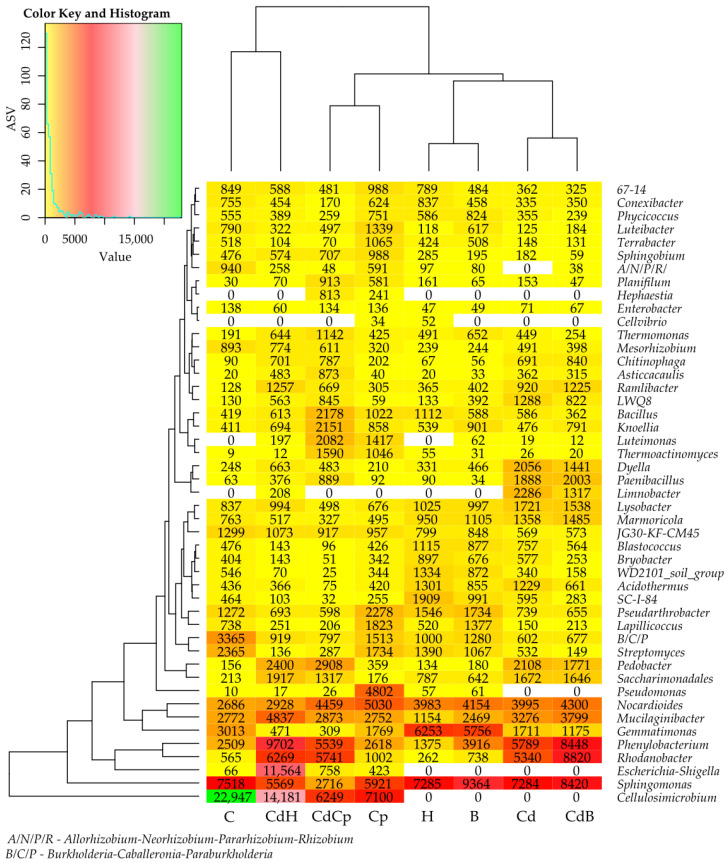
Dominant bacterial genera in uncontaminated and cadmium-contaminated soils. C—uncontaminated soil, Cp—uncontaminated soil with compost, B—uncontaminated soil with fermented bark, H—uncontaminated soil with HumiAgra, Cd—soil contaminated with Cd^2+^, CdCp—soil contaminated with Cd^2+^ and compost, CdB—soil contaminated with Cd^2+^ and fermented bark, CdH—soil contaminated with Cd^2+^ and HumiAgra.

The genus-level analysis (Figure 3) showed that *Sphingomonas* was the most abundant genus. It was most abundant after application of B in both uncontaminated soil (9364 ASV) and cadmium-contaminated soil (8420 ASV). The lowest abundance was observed after application of Cp in uncontaminated soils (5921 ASV) and contaminated soils (2716 ASV). Bacteria of the genus *Phenylobacterium* were also highly abundant. Their abundance, however, depended on cadmium exposure (CdH: 9702 ASV > CdB: 8448 ASV > Cd: 5539 ASV = CdCp: 5539 ASV). Bacteria of the genera *Nocardioides* and *Mucilaginibacter* were also highly abundant in all soil samples.

Changes in bacterial composition were observed in *Gemmatimonas*, with ASV counts in soil samples after the application of H being the highest (6253 ASV) and B (5756 ASV). They accounted for over 6.6% of all ASV counts. In contrast, in cadmium-contaminated soil, their abundance decreased to 1711 ASVs, accounting for less than 2% of the total ASVs. *Pseudarthrobacter*, *Streptomyces*, and *Burkholderia-Caballeronia-Paraburkholderia* also reacted very similarly to Cd^2+^. It was found that the addition of Cp most significantly reduced the cadmium-induced negative impact on *Cellulosimicrobium*. H exhibited the second strongest effect on *Cellulosimicrobium*.

**Figure 4:**
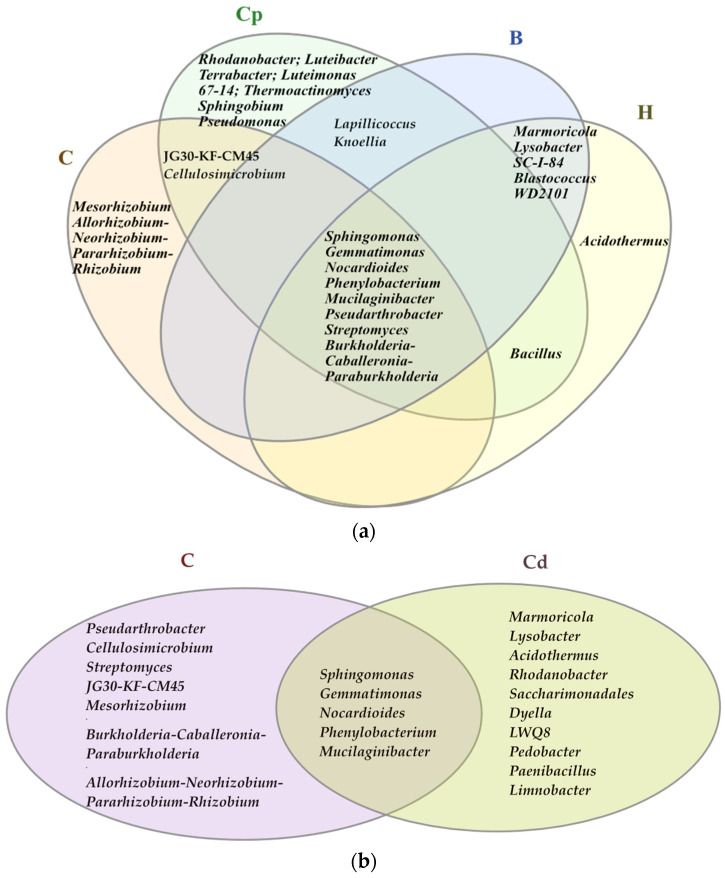

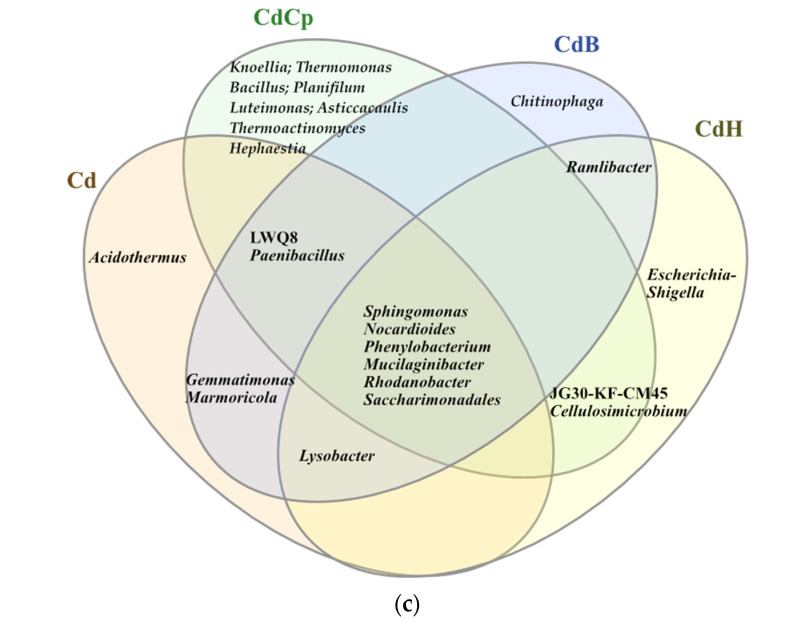
Common and unique bacterial genera: in soil enriched with organic matter (**a**); in uncontaminated and cadmium-contaminated soil (**b**); in cadmium-contaminated soil enriched with organic matter (**c**). C—uncontaminated soil, Cp—uncontaminated soil with compost, B—uncontaminated soil with fermented bark, H—uncontaminated soil with HumiAgra, Cd—soil contaminated with Cd^2+^, CdCp—soil contaminated with Cd^2+^ and compost, CdB—soil contaminated with Cd^2+^ and fermented bark, CdH—soil contaminated with Cd^2+^ and HumiAgra.

The foundation of this study was the identification of key information regarding the composition of the soil bacterial microbiome (Figure 4). To precisely identify unique and shared bacteria characteristic of the individual study treatments, an analysis of ASVs ≥ 1% was conducted separately for uncontaminated soil, cadmium-contaminated soil, and soil contaminated with organic additives. The use of Venn diagrams enabled the identification of common taxa, referred to as the core microbiome, and those whose presence was strictly determined by organic amendments. Thus, in uncontaminated soil with humic acids, the unique bacterial genus was *Acidothermus*; with compost: *Rhodanobacter*, *Luteibacter*, *Terrabacter*, *Luteimonas*, 67-14, *Thermoactinomyces*, *Sphingobium*, and *Pseudomonas*; and in the control soil (C), bacteria of the genera *Mesorhizobium* and *Allorhizobium-Neorhizobium-Pararhizobium-Rhizobium*. Importantly, no unique bacterial genera were identified in the uncontaminated soil after the application of fermented bark. The genera common to all uncontaminated soils were: *Sphingomonas*, *Gemmatimonas*, *Nocardioides*, *Phenylobacterium*, *Mucilaginibacter*, *Pseudarthrobacter*, *Streptomyces*, *Burkholderia*, *Caballeronia*, and *Paraburkholderia*. Cadmium altered the structure of the soil bacterial microbiome. In cadmium-contaminated soil, regardless of organic matter supplementation, the common bacterial microbiome consisted of: *Sphingomonas*, *Nocardioides*, *Phenylobacterium*, *Mucilaginibacter*, *Rhodanobacter*, and *Saccharimonadales*.

### 2.3. Bacterial Diversity

**Figure 5:**
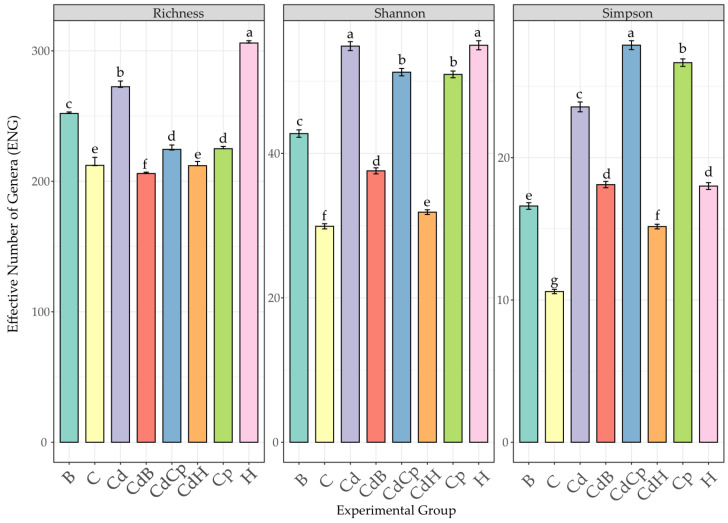
Alpha diversity of bacteria expressed as the effective number of genera (ENG). Homogeneous groups marked with letters (a–g) were calculated separately for each diversity index. C—uncontaminated soil, Cp—uncontaminated soil with compost, B—uncontaminated soil with fermented bark, H—uncontaminated soil with HumiAgra, Cd—soil contaminated with Cd^2+^, CdCp—soil contaminated with Cd^2+^ and compost, CdB—soil contaminated with Cd^2+^ and fermented bark, CdH—soil contaminated with Cd^2+^ and HumiAgra.

To compare biodiversity among the studied treatments, an alpha diversity analysis was performed based on Hill numbers (Figure 5, Table S1), where the effective number of bacterial genera (ENG) was calculated for three diversity orders: richness (q = 0), Shannon diversity (q = 1), and Simpson diversity (q = 2) (Hill numbers [27]). The alpha diversity analysis revealed significant differences in community structure among the soil samples, as the observed number of genera (S_oβs_) closely matched the estimated total bacterial diversity across all treatments. It was found that the highest richness, and thus the highest values of the effective number of genera (ENG), were observed in soil treated with humic acids (306.00 ± 0.9) and in cadmium-contaminated soil (272.50 ± 2.23). The lowest ENG values were recorded in Cd^2+^-contaminated soil following the application of fermented bark (206.00 ± 0.52) and humic acids (212.00 ± 1.65). The Shannon diversity index, which accounts for dominance structure, showed the highest values in the H and CdB treatments, as did the richness index. Importantly, in uncontaminated soil (C), despite high richness (q = 0), the ENG q = 1 index decreased sharply. Simpson’s diversity analysis (q = 2), however, indicated that the CdCp and Cp treatments had the most balanced structure (the highest ENG values: 27.90 ± 0.16 and 26.67 ± 0.13, respectively). Analysis of higher-order indices (q = 1 and q = 2) revealed that, in our study, richness did not always translate into high structural diversity.

**Figure 6:**
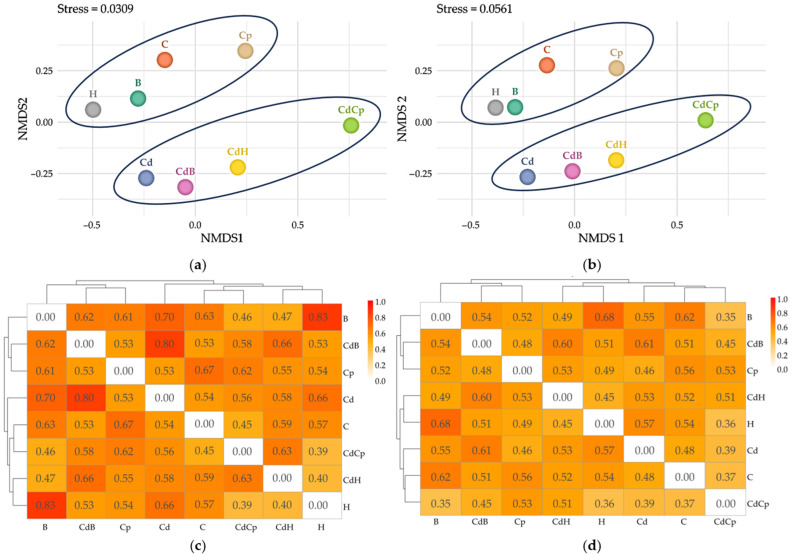
Beta diversity of bacteria, expressed as the Bray–Curtis dissimilarity index (**a**,**c**) and the Jaccard dissimilarity index (**b**,**d**), presented on NMDS (Non-metric Multidimensional Scaling) plots and heatmaps. C—uncontaminated soil, Cp—uncontaminated soil with compost, B—uncontaminated soil with fermented bark, H—uncontaminated soil with HumiAgra, Cd—soil contaminated with Cd^2+^, CdCp—soil contaminated with Cd^2+^ and compost, CdB—soil contaminated with Cd^2+^ and fermented bark, CdH—soil contaminated with Cd^2+^ and HumiAgra.

To assess the *β*-diversity of bacterial communities among the studied treatments, Bray–Curtis analysis (based on bacterial community abundance) (Figure 6a,c) and Jaccard analysis (based on the presence or absence of individual genera) (Figure 6b,d) were performed. The data were visualized using non-metric multidimensional scaling (NMDS) and heatmaps (Figure 6a–d). The Bray–Curtis scaling method used was characterized by very good representation of the results (stress = 0.0309), which allowed for the identification of the most unique composition of bacterial genera in the CdCp treatment (NMDS1: 0.76; NMDS2: −0.02), an extremely different microbiome in the H treatment compared to CdCp, and the most similar to each other in the Cd^2+^, CdB, and CdH soils (Figure 6a). The distribution of Bray–Curtis dissimilarity indices on a heat map (Figure 6c) reveals high indices in treatments B and H (0.83) and CdB and Cd^2+^ (0.80). The lowest indices were observed in the CdH and H soils (0.39) and the CdCp and H soils (0.40).

The Jaccard analysis (Figure 6b), which considers only the presence or absence of individual taxa, showed a pattern similar to that of the Bray–Curtis analysis. The stress value (0.056) indicates good representation of the data in the ordination space, confirming the general trend observed in the Bray–Curtis analysis. However, a difference in bacterial dissimilarity was observed between the Cd^2+^ and CdCp soil samples. In the Jaccard analysis, the NMDS1 value was 0.64, while in the Bray–Curtis analysis, it was 0.76. Thus, the CdCp soil most likely contains unique bacterial species not found in the other soil samples. The calculated Jaccard indices (Figure 6d) were lower than the Bray–Curtis indices (Figure 6c). The highest Jaccard indices were found in the soils between B and H (0.68); B and C (0.62); and CdB and Cd^2+^ (0.61), while the lowest occurred between B and CdCp (0.35).

### 2.4. Metabolic and Ecological Functions of Bacteria

**Figure 7:**
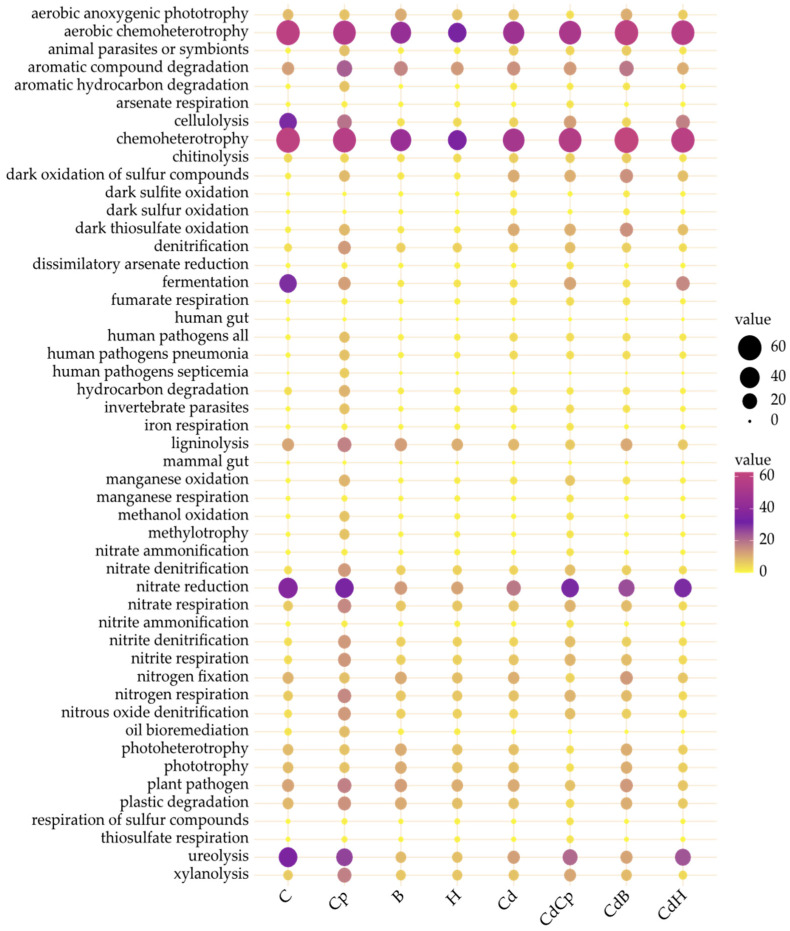
Known and predicted metabolic functions of bacteria (16S rRNA—ASV) assigned based on their genus, %. C—uncontaminated soil, Cp—uncontaminated soil with compost, B—uncontaminated soil with fermented bark, H—uncontaminated soil with HumiAgra, Cd—soil contaminated with Cd^2+^, CdCp—soil contaminated with Cd^2+^ and compost, CdB—soil contaminated with Cd^2+^ and fermented bark, CdH—soil contaminated with Cd^2+^ and HumiAgra.

An analysis performed using MACADAM software (https://github.com/maloleboulch/MACADAM-Database, accessed on 24 June 2026) [28] indicated that chemoheterotrophs and aerobic chemoheterotrophs predominated in all analyzed soil samples (Figure 7), accounting for 34% to 63%. These were bacteria of the genera *Acidothermus*, *Blastococcus*, *Terrabacter*, *Asticcacaulis*, *Mesorhizobium*, *Bacillus*, *Rhodanobacter*, *Streptomyces*, *Marmoricola*, *Sphingobium*, *Cellvibrio*, *Nocardioides*, *Limnobacter*, *Dyella*, *Sphingomonas*, *Pedobacter*, *Paenibacillus*, *Phenylobacterium*, *Thermomonas*, *Hephaestia*, *Conexibacter*, *Mucilaginibacter*, *Phycicoccus*, *Gemmatimonas*, *Cellulosimicrobium*, *Pseudomonas*, *Knoellia*, *Lapillicoccus*, *Bryobacter*, and *Thermoactinomyces*, *Enterobacter*, *Lysobacter*, and *Chitinophaga* (Table S2). Most of the identified bacterial genera were dominant in the studied soils, except for *Cellvibrio*, *Conexibacter*, *Phycicoccus*, *Bryobacter*, and *Enterobacter*. Among the key metabolic functions of bacteria, aromatic compound degradation was also significant, with its contribution to the bacterial microbiome ranging from 11% (CdH) to 23% (Cp). Bacteria were responsible for nitrate reduction ranging from 12% (H) to 38% (C). The highest percentage of bacteria responsible for this process was recorded in treatments C and Cp, followed by CdCp, CdH, and CdB. The urea-degrading activity of the bacteria ranged from 8% to 35%, with the highest values observed in treatment C, followed by Cp, CdH, CdCp, Cd, CdB, B, and H. In turn, the proportion of functions related to cellulose degradation ranged from 3% (CdCp) to 30% (C).

The significant dominance of carbon cycle-related processes among chemoheterotrophs and aerobic chemoheterotrophs was most strongly associated with the abundance of *Cellulosimicrobium*, *Gemmatimonas*, *Mucilaginibacter*, *Nocardioides*, *Phenylobacterium*, *Sphingomonas*, *Rhodanobacter*, and *Streptomyces*. These constituted the majority of the shared microbiome of both uncontaminated and cadmium-contaminated soils. Among bacteria assigned to these functional categories, *Cellulosimicrobium* was particularly abundant and represented a major component of the soil microbial community (Table S2). The convergence of results for both of these functions was confirmed by the processes of organic matter oxidation under aerobic conditions, which are the dominant metabolic pathway in the analyzed soil. The key genera identified in the nitrate respiration category were again *Cellulosimicrobium*, *Nocardioides*, *Rhodanobacter*, and *Pseudomonas*, while urea hydrolysis was primarily driven by *Cellulosimicrobium*, *Pedobacter*, and *Pseudomonas*.

**Figure 8:**
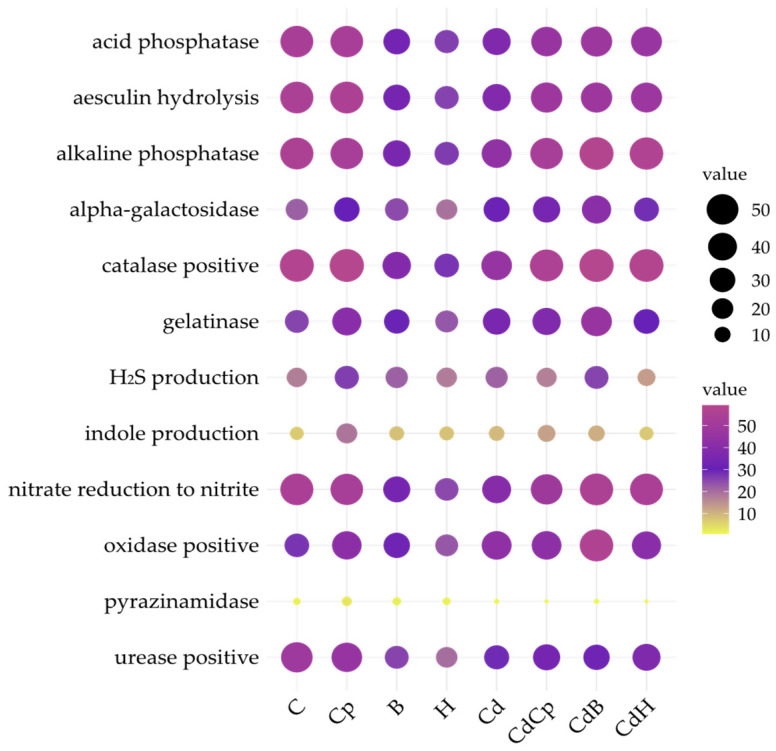
Metabolic potential of the soil bacterial microbiome, %. C—uncontaminated soil, Cp—uncontaminated soil with compost, B—uncontaminated soil with fermented bark, H—uncontaminated soil with HumiAgra, Cd—soil contaminated with Cd^2+^, CdCp—soil contaminated with Cd^2+^ and compost, CdB—soil contaminated with Cd^2+^ and fermented bark, CdH—soil contaminated with Cd^2+^ and HumiAgra.

An analysis of the functional potential of the bacterial community revealed a range of metabolic functions (Figure 8), related to carbon, nitrogen, and phosphorus metabolism. In most treatments, the highest bacterial potential was associated with aerobic respiration (catalase-positive), phosphorus metabolism (acid phosphatase and alkaline phosphatase), and the reduction of nitrate (NO_3_^−^) to nitrite (NO_2_^−^). In the control soils (C and Cp), acid phosphatase activity was 54% and 53%, respectively, whereas alkaline phosphatase activity was 55% and 53%. Lower values were observed in soils B and H, where acid phosphatase activity was 34% and 26%, respectively, and alkaline phosphatase activity was 36% and 27%. In soils under Cd^2+^ stress, the proportion of bacteria responsible for these functions was higher than in treatments B and H, but lower than in soils C and Cp. In the case of acid phosphatase, bacterial populations were responsible for 38% of the enzyme activity in Cd, 46% in CdCp, 48% in CdB, and 47% in CdH. A similar trend was observed for alkaline phosphatase, with values of 44% in Cd, 53% in CdCp, 58% in CdB, and 57% in CdH, respectively.

Similarly, the bacterial microbiome exhibited high functional potential for nitrogen transformation processes (nitrate reduction to nitrite), reaching 55% in C, 53% in Cp, 34% in B, and 25% in H. In Cd^2+^-contaminated soils, these values were 49% in CdCp, 55% in CdB, and 54% in CdH. 49% for CdCp, 55% for CdB, and 54% for CdH. A substantial bacterial contribution to urease activity was observed. In the C and Cp treatments, it accounted for 49% and 46%, respectively, whereas in the B and H treatments, it was 26% and 20%, respectively. In Cd^2+^-contaminated soils, bacterial contributions to urease activity were 35% in CdCp, 32% in CdB, and 37% in CdH. Oxidase-positive bacteria accounted for 56% in CdB, 43% in Cd, 42% in CdCp, 41% in Cp, and 41% in CdH, respectively. The lowest values were recorded in B (33%) and H (23%).

The identified functions included enzymatic activities associated with the mineralization of organic compounds, such as alpha-galactosidase, gelatinase, and aesculin hydrolysis, indicating the microorganisms’ potential to degrade complex organic compounds. The aesculin hydrolysis function showed a similar bacterial pool as for phosphatase activity in C and Cp (55% in both cases). In the treatments B and H, these values were lower, at 34% and 26%, respectively. In soils with added Cd^2+^, the population of bacteria responsible for this process increased to 38% (Cd), 49% (CdCp), and 48% (CdB and CdH). Significantly fewer bacterial communities were responsible for the production of alpha-galactosidase, with values ranging from 19% (H) to 41% (CdB), and gelatinase, ranging from 23% (H) to 46% (CdB).

The lowest functional potential of the bacterial microbiome under study was observed for processes linked to hydrogen sulfide (13–26%) and indole synthesis (7–19%). In addition, the presence of the enzyme pyrazinamidase was identified; however, the bacterial pool responsible for producing this enzyme was negligible (1–3%). This analysis also revealed the presence of bacterial genera with high metabolic plasticity, exhibiting activity across a broad spectrum of enzymatic processes. Key representatives included the genera *Rhodanobacter*, *Nocardioides*, *Sphingomonas*, *Mucilaginibacter*, and *Cellulosimicrobium* (Table S3), which dominated both hydrolytic processes (such as acid and alkaline phosphatase activities, esculin hydrolysis, and alpha-galactosidase) and nitrogen metabolism (including nitrate reduction to nitrite and ureolysis). Bacteria of the genus *Pseudomonas* exhibited strong biochemical potential, displaying activity in most of the tested processes, including gelatinase production, as well as catalase and oxidase activity. The co-occurrence of these traits in dominant taxa suggests their key role in stabilizing biochemical processes and biogeochemical cycles of C, N, and P. Such accumulation of functions within the same bacterial genera (particularly *Rhodanobacter* and *Nocardioides*) indicates functional redundancy.

**Figure 9:**
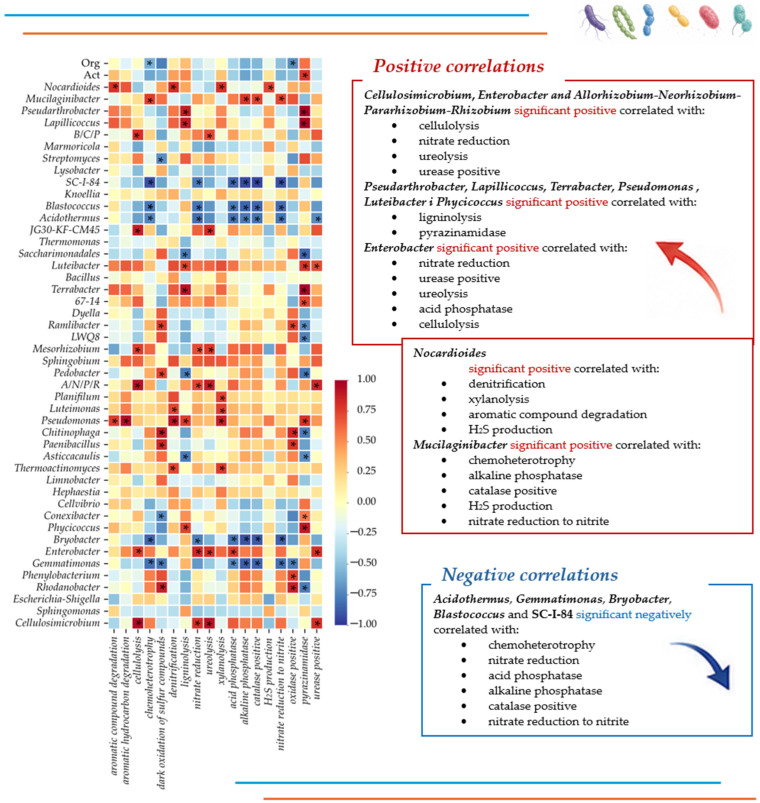
Pearson correlation coefficients between bacterial types and selected metabolic functions. *—statistically significant (*p* < 0.05).

The results of the study indicate clear functional differences between bacterial genera (Figure 9). Bacteria of the genera *Cellulosimicrobium*, *Enterobacter*, and *Nocardioides* were significantly positively correlated with processes related to the decomposition of organic matter, nitrogen and phosphorus cycling, and the transformation of complex organic substrates. Bacteria of the genera *Acidothermus*, *Blastococcus*, SC-I-84, and *Gemmatimonas* showed a significant negative correlation with selected hydrolytic and oxidative enzyme activity, as well as with chemoheterotrophy and nitrate reduction. Furthermore, significant positive correlations were observed between selected bacterial genera and functions related to organic polymer degradation, ligninolysis, cellulolysis, pyrazinamidase activity, and sulfur oxidation. The obtained research results thus indicate the diversity of ecological niches and metabolic potential of bacteria inhabiting the studied soil and may reflect their complementary role in the functioning of the soil environment.

## 3. Discussion

Since the soil bacterial microbiome is essential for maintaining soil fertility and biogeochemical cycling, cadmium-induced disturbances in its structure and activity can adversely affect soil functioning [2,29]. Therefore, organic amendments are increasingly being explored as sustainable approaches for mitigating heavy metal stress. Their effectiveness results not only from the immobilization of contaminants but also from their ability to shape the soil microbial environment and support microbial activity [2,29,30,31,32,33]. In uncontaminated soil (C), the bacterial community structure reflected stable ecological conditions and a well-balanced nitrogen cycle. The diversity of the bacterial soil microbiome observed in uncontaminated soil clearly highlights the role of the quality and availability of exogenous organic matter as a key selective factor determining ecological niches. In the control soil (C), the dominance of typical diazotrophic and rhizosphere bacteria, such as *Mesorhizobium* and the *Allorhizobium-Neorhizobium-Pararhizobium-Rhizobium*, indicates a natural, stable nitrogen cycle and optimal conditions conducive to the development of the endogenous microbiome of plant-growth-promoting bacteria (PGPB).

The introduction of humic acids (H) led to a marked restructuring of the community, with the genus *Acidothermus* becoming dominant. This phenomenon can be explained by the fact that humic acids constitute a pool of carbon that is highly resistant to rapid decomposition. As indicated by the latest studies by Garbacz et al. [34], Kamal et al. [35], Peng et al. [36], Datta et al. [37], and Li et al. [38], this promotes the development of specialized oligotrophs possessing an extensive enzymatic apparatus capable of metabolizing complex polymers and tolerating specific changes in physicochemical properties, including potential local pH reductions associated with these acids. A completely different dynamic was observed following the application of compost (Cp), which, by supplying significant amounts of readily available nutrients, triggered a rapid proliferation of r-strategy bacteria, known as copiotrophs [39,40,41]. The high abundance of bacteria belonging to the genera *Pseudomonas*, *Sphingobium*, *Rhodanobacter*, which are known for their high denitrification activity in carbon-rich environments, as well as *Luteimonas*, represents a typical rapid environmental response to the input of labile organic matter. Additionally, the presence of genera such as *Thermoactinomyces* and *Terrabacter* is most likely a direct result of the carry-over effect, reflecting the survival and adaptation of the native bacterial community in the compost.

Despite the described structural shifts induced by organic amendments, the identification of the so-called core microbiome—consistently comprising *Sphingomonas*, *Gemmatimonas*, *Nocardioides*, *Phenylobacterium*, *Mucilaginibacter*, *Pseudarthrobacter*, *Streptomyces*, and the *Burkholderia-Caballeronia-Paraburkholderia* complex—in all uncontaminated soils attests to the ecosystem’s exceptionally high stability. These metabolically versatile taxa exhibit broad plasticity in utilizing diverse carbon sources and provide functional redundancy for key biogeochemical processes, thus maintaining soil homeostasis.

Cadmium contamination in the soil created a strong selective pressure, altering the microbiome structure, reducing its original diversity, and favoring bacteria that tolerate oxidative stress and cadmium toxicity. The decrease in dominant groups such as *Actinomycetota* and the increase in fast-growing r-strategist taxa (*Pseudomonadota* and *Bacteroidota*) may account for the higher values of taxonomic richness indices in contaminated soils compared to the control soil. The toxic effect of cadmium weakens competition from dominant taxa, thereby enabling the growth and detection of rarer or more environmentally responsive bacteria. These results are consistent with the observations of Xu et al. [42] and Yang et al. [43], who demonstrated that cadmium leads to a decrease in the abundance of some dominant taxa and an increase in the proportion of rare taxa. Despite such severe perturbation in our own studies, a core microbiome consisting of the genera *Sphingomonas*, *Mucilaginibacter*, *Nocardioides*, *Phenylobacterium*, and *Gemmatimonas* was still observed. Their widespread presence in cadmium-contaminated soils indicates the possession of universal stress adaptation mechanisms [44], which have been observed in *Mucilaginibacter* [14], *Sphingomonas* and *Agrobacterium* [33,45], *Pseudomonas* [33,44,45,46], *Bacillus* [44], *Nocardioides* [47], *Phenylobacterium* [48] and *Gemmatimonas* [49]. They may also produce exopolysaccharides (EPS), which form a biofilm that binds and immobilizes free heavy metal ions (including Cd^2+^) in the extracellular space, protecting cells from metal penetration. According to the aforementioned researchers, strains of these bacteria also secrete EPS, thereby contributing to improved soil aggregate stability and increased water and nutrient retention [50]. In turn, *Rhodanobacter* is a taxon known for its extreme tolerance to low pH and high metal concentrations [51,52], which may indicate its significant role in maintaining essential biogeochemical functions in degraded environmental niches.

Against the backdrop of the core microbiome, the diversity of organic additives created unique pathways of microbial succession. Exceptionally interesting dynamics were observed in soils with humic acids (CdH), where *Acidothermus* emerged as the dominant and unique genus, present in both uncontaminated and cadmium-contaminated soils with H. Bacteria of this genus possess the ability to decompose organic carbon compounds resistant to degradation. They also produce thermostable enzymes, as noted in the study by Li et al. [38]. Importantly, it was also demonstrated that strains of this genus can degrade mono- and polycyclic aromatic compounds, which suggests their high efficacy in soil remediation. The identified cellulose-degrading *Acidothermus* strains and phenolic acid-degrading *Sphingomonas* strains [38] may exhibit potential “synergistic” or “antagonistic” effects within the microecosystem. According to Tang et al. [53], humic compounds are also weakly bound to extracellular polymeric substances (EPS), whereas according to Michalska et al. [54], EPS can act as a “sponge” for various organic pollutants and undergo hydrolysis only as organic matter (OM) particles and cellular materials present in the structure degrade. Research by Kong et al. [55], however, suggests that under stress conditions (cadmium toxicity), humic acids significantly alter the microenvironment around cells. They act as electron shuttles, influence the secretion of extracellular polymeric substances (EPS), and bind to minerals and cell surfaces, forming a molecular barrier. This suggestion may therefore explain the dominance of the acidophilic *Acidothermus*, as it is precisely these bacteria, having access to H, that were able to manage accumulated energy more efficiently under the extremely harsh conditions induced by Cd^2+^, which gives them a competitive advantage over sensitive bacteria. The remaining core bacterial microbiome, including *Sphingomonas* and *Mucilaginibacter*, survived cadmium contamination; both are known to produce exopolysaccharides that protect the soil from adverse physicochemical and biological conditions. Simultaneously, the same studies demonstrated a significant proliferation of bacteria in the genera *Escherichia* and *Shigella*, suggesting an opportunistic adaptation strategy in these copiotrophic taxa, driven by the utilization of readily available carbon fractions released through interactions with humic acids under contaminated conditions [56,57,58].

A completely different selection mechanism emerged after applying fermented bark (CdB), with bacteria of the genus *Chitinophaga* showing unique dominance. As a genus specialized in degrading complex biopolymers, including cellulose and chitin [59,60,61], these bacteria likely utilized the lignocellulosic matrices of the bark both as a carbon source and as a physical protective barrier against cadmium toxicity.

In contrast, the application of compost (CdCp treatments) most effectively buffered cadmium toxicity, enabling the survival and growth of bacterial genera such as *Knoellia*, *Thermomonas*, *Bacillus*, *Planifilum*, *Luteimonas*, *Asticcacaulis*, *Thermoactinomyces*, and *Hephaestia*. The presence of taxa capable of forming spore-forming structures, e.g., *Bacillus* and *Thermoactinomyces*, as well as highly thermophilic bacteria typical of the late stages of composting (*Thermoactinomyces*, *Thermomonas*, *Planifilum*), demonstrates a strong inoculation effect. Compost not only immobilized cadmium by introducing rich organic matter but also acted as a vector transferring a highly resistant bacterial microbiome (*Thermomonas*, *Bacillus*, *Luteimonas*, *Thermoactinomyces*). These taxa successfully colonized the contaminated soil and adapted to stressful conditions. The results of our study are consistent with those of Lahori et al. [62], and Chiodi et al. [63], who analyzed the potential of compost to fix heavy metals and increase soil nutrient content. Similarly, the study by Chiarelli et al. [64] showed that compost from municipal organic waste increased the abundance of bacteria of the genus *Planifilum* and *Bacillus*, while compost from agricultural and food waste promoted the genus *Chitinophaga*.

Analysis of alpha and beta diversity confirmed a significant effect of Cd^2+^ contamination on soil bacterial diversity. According to Xu et al. [65] and Sun et al. [66], one possible reason for the bacterial response to Cd^2+^ is that soil microorganisms adapt to stress by regulating their metabolism; they may alter population diversity by exerting selective pressure, but may also leave other populations unaffected. Changes in bacterial diversity were also observed following the application of organic sorbents, as confirmed by studies by Rodríguez-Berbel et al. [67], Kraut-Cohen et al. [68] and Zhao et al. [69]. Kraut-Cohen et al. [68] demonstrated that the application of compost significantly increased bacterial alpha diversity and caused marked shifts in the structure of the soil microbiome, particularly in beta diversity. The changes were dependent on the compost dose and the time of application. According to Zhao et al. [69], the composting process leads to marked changes in the structure of microbial communities, accompanied by reductions in humic substances, the molecular weight of humic acids, and their chemical structure. Thus, the composition of organic matter and the degree of humification may act as a selective factor determining the composition of bacterial communities. The studies by Rousseau et al. [70] confirm our findings regarding the smallest changes in diversity following the application of B. The authors demonstrated that increased amounts of dead wood did not significantly affect the alpha diversity of bacteria and other soil microorganisms. Species richness and overall microbial diversity thus remained relatively stable despite the increased influx of organic matter. At the same time, clear changes in beta diversity and bacterial community composition were observed among the studied treatments.

Functional profiling of soil bacterial communities enabled assessment of ecological processes modified by organic amendments in uncontaminated and cadmium-contaminated soils. The most abundant functional groups were chemoheterotrophy and aerobic chemoheterotrophy, which are characteristic of environments rich in organic carbon [71,72]. In soils amended with B and H, a significant decrease in the proportion of these functions was observed compared with C and Cp. This indicates a shift from nonspecific carbon degradation toward more specialized metabolic processes, including nitrate reduction and urea transformation. These patterns are consistent with our previous observations showing the dominance of chemoheterotrophic bacteria primarily involved in nitrogen cycling and organic matter decomposition in cadmium-stressed soil supplemented with humic acids in the form of the HumiAgra preparation [72].

In the study by Chiodi et al. [63], a significant increase in nitrification processes was observed, accompanied by a decrease in organic carbon degradation following the application of compost at 2% and 5%. The observed functional changes thus confirm that organic amendments can restructure the soil microbiome toward functions involved in the biogeochemical cycling of elements essential for plant growth and development. This also underscores the importance of compost, humic acids, and fermented bark in improving soil fertility, particularly under heavy metal stress.

The functional metabolic predictions presented in this study should be interpreted with caution. Predictive tools rely heavily on available reference genomes and databases, which may lead to inaccurate estimates. Approaches based on 16S rRNA gene sequencing cannot capture strain-level metabolic variability or horizontal gene transfer, both of which play crucial roles in microbial adaptation and functional diversity. As highlighted by Matchado et al. [73], shotgun metagenomics represents a more comprehensive and appropriate approach for assessing the metabolic potential of microbiomes. Nevertheless, our predictive data provide valuable preliminary insights into metabolic alterations occurring within the investigated environment. Future studies employing shotgun metagenomics or metatranscriptomics are required to conclusively validate these functional profiles.

**Figure 10:**
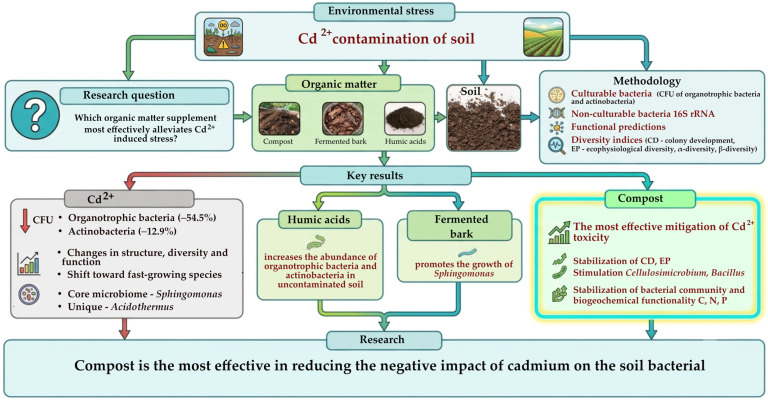
Graphical summary of the research project.

In summary, it should be emphasized that Cd^2+^-contaminated soil disrupted both the abundance and structure of the bacterial community (Figure 10). The soil applied organic amendments modulated the response of the bacterial microbiome to cadmium-induced stress, with compost being the most effective in mitigating its adverse effects and stabilizing the bacterial community. Thus, compost proved to be the most suitable amendment for the remediation of cadmium-contaminated soil.

## 4. Materials and Methods

### 4.1. Soil Sampling and Characterization

The study was conducted on sandy loam (SL) soil with 63.61% sand, 32.68% silt, and 3.71% clay, and organic carbon and nitrogen contents of 10 g C_org_ kg^−1^ and 0.83 g N_tot_ kg^−1^ d.m. of soil, respectively. The soil was acidic, with a pH of 4.40 in 1 mol KCl dm^−3^ and 5.52 in H_2_O. Exchangeable base cations amounted to 63.60 mM(^+^) kg^−1^ d.m. of soil, cation exchange capacity was 89.70 mM(^+^) kg^−1^ d.m. of soil and alkaline cation saturation was 70.90%. Before the experiment began, the soil was dried at room temperature, ground, and sieved through a 5 mm mesh sieve to remove plant debris and larger mineral particles. A portion of the soil was contaminated with cadmium at a level corresponding to 15 mg Cd^2+^ kg^−1^ d.m. of soil. Soil remediation was carried out using organic substances, i.e., compost (Cp), fermented bark (B), and the organic product HumiAgra (H). The compost was prepared in-house from grasses and had the following composition, in g kg^−1^ d.m. of soil: C_org_—146.61 and N_tot_—20.18, P—3.41, K—9.25, and Mg—5.69. The soil pH, measured in 1 mol KCl dm^−3^, was 6.1. The fermented bark, with a diameter of approximately 20–50 mm, was prepared from coniferous tree bark (Athena Bio-Produkty Sp. z o.o., Golczewo, Poland). It was characterized by a dry matter content of ≥30% and an organic matter content of ≥50%. Its pH in KCl was 3.82. HumiAgra, a dark brown powder containing 90% humic acids, with a humic-to-fulvic acid ratio of 1:1, contained 8% K_2_O and 3% S (AgraPlant, Kielce, Poland). Its pH ranged from 8 to 10. The test plant was *Zea mays* variety DS1897B, which was described in detail in our previous study [9].

### 4.2. Experimental Design

The experiment was conducted in a greenhouse in northeastern Poland. The study was carried out in polyethylene pots measuring 16 cm in height with a base diameter of 17 cm. The experiment was divided into two treatments: (1) control soil (without Cd^2+^) and (2) soil contaminated with Cd^2+^. Cadmium was applied as 3CdSO_4_·8H_2_O (Sigma-Aldrich, Saint Louis, MO, USA). Various soil-improving materials were introduced into both series at a rate of 3 g C kg^−1^ of soil. These included compost (Cp), fermented bark (B), product HumiAgra (H), and mineral fertilization to ensure adequate availability of essential nutrients for plants. Nitrogen was applied as urea [CO(NH_2_)_2_], phosphorus as potassium phosphate [KH_2_PO_4_], and potassium and magnesium were supplied as mineral salts [KH_2_PO_4_ + KCl and MgSO_4_·7H_2_O] (STANLAB, Lublin, Poland). The application rates were 150 mg N kg^−1^ of soil, 50 mg P, 150 mg K, and 20 mg Mg. Each experimental treatment was conducted in four replicates. Soil moisture was maintained at 60% of the soil’s maximum water-holding capacity throughout the 55-day experiment. Four plants were grown in each pot. The experiment was conducted in June and July 2022. In 2022, the mean annual air temperature in Olsztyn (Poland) was 8.9 °C. In June 2022, the mean daily temperature was 17.9 °C, with maximum and minimum values of 24.1 °C and 11.9 °C, respectively. In July 2022, the corresponding values were 18.0 °C for the mean temperature, 23.5 °C for the maximum temperature, and 13.2 °C for the minimum temperature [74]. On the day of harvest, the entire soil from each pot was transferred to a sterile container, the roots were removed, and the soil was thoroughly mixed to obtain a homogeneous sample. Immediately after collection, the soil samples were stored at −32 °C. Microbiological analyses were initiated within 5 days of soil sample collection.

### 4.3. Cultivable Bacteria

The abundance of organotrophic bacteria and actinobacteria was expressed as CFU kg^−1^ of d.m. of soil. The isolation procedure was performed using the deep plating technique with a series of soil dilutions. To obtain reliable results, the analysis was performed in four replicates for each sample. Organotrophic bacteria were cultured on Bunt and Rovira medium, while Küster and Williams medium was used for actinobacteria. The inoculated plates were incubated for ten days at 28 °C in a PSelecta Incudigit laboratory incubator (Barcelona, Spain). The number of colonies of both microbial groups was systematically counted at 24 h intervals for 10 consecutive days. These data were used to calculate the colony development index (CD) according to the method proposed by Sarathchandra et al. [75] and the ecophysiological diversity index (EP) described by De Leij et al. [76].

### 4.4. DNA Extraction and 16S rRNA Amplicon Sequencing

The material for microbiological analysis consisted of soil samples collected after the end of the growing season. Immediately after collection, DNA was isolated from approximately 0.5 g of fresh soil using the Genomic Mini AX Bacteria+ kit (A&A Biotechnology, Gdynia, Poland). To improve cell lysis efficiency, samples were mechanically homogenized using a FastPrep-24 device with zirconia beads. The resulting DNA extracts were further purified using the Anti-Inhibitor Kit (A&A Biotechnology, Gdynia, Poland), which removed potential inhibitors of enzymatic reactions. The concentration of the isolated DNA was determined by fluorometric analysis using a Qubit 4 Fluorometer (Thermo Fisher Scientific, Waltham, MA, USA). The presence of bacterial DNA in the samples was confirmed by quantitative real-time polymerase chain reaction (qPCR). The reactions were performed in a CFX Connect thermal cycler (BioRad Laboratories, Hercules, CA, USA) using SYBR Green dye as a fluorochrome. Amplification of the 16S rRNA gene fragment was performed using the universal primers 341F (5′-CCTACGGGNGGCWGCAG-3′) and 785R (5′-GACTACHVGGGTATCTAATCC-3′), commonly used for bacterial detection [77]. The PCR reaction was performed using the Q5 Hot Start High-Fidelity 2X Master Mix (New England Biolabs, Ipswich, MA, USA), and sequencing was carried out on a MiSeq instrument using paired-end (PE) technology (2 × 300 nt) with the Illumina v3 kit (Genomed, Warsaw, Poland).

### 4.5. Bioinformatic Analysis

Read alignment was performed using QIIME 2 software [78] with the SILVA 138 reference sequence database [79,80,81]. Raw sequence data underwent preliminary quality control and filtering to remove low-quality reads and fragments that did not meet the length criteria. Adapter sequences and low-quality fragments were removed prior to further analysis. Sequencing errors were corrected using the DADA2 algorithm [82], and optimal parameters for paired-read trimming were determined using the FIGARO tool [83]. The bioinformatics analysis was performed by the certified Genetic Diagnostics Laboratory of Genomed SA, Poland. Microbial sequences were submitted to the National Center for Biotechnology Information under GenBank accession numbers: prokaryotic 16S rRNA: https://www.ncbi.nlm.nih.gov/nuccore/?term=PZ180090-PZ182063 (accessed on 18 March 2026).

### 4.6. Statistical Analysis

Statistical analyses were performed in the R computing environment within RStudio (Posit Software, Boston, MA, USA, version 2023.06.0 Build 421) (using packages designed for microbiological data analysis. The effects of organic amendments and cadmium-induced stress on the diversity and structure of cultured and uncultured bacterial communities were assessed using ANOVA (Statistica 13.3) [84]. Homogeneous groups were identified for all independent variables using Duncan’s test. Differences between treatment variants were considered statistically significant at *p* < 0.05. To determine alpha diversity and beta diversity, we used the R environment (v. 2026.1.2.418), the iNEXT package (v. 3.0.2), vegan (v. 2.7.3), and ggplot2 (v. 4.0.2). Alpha diversity results are presented as Hill numbers [85,86], expressed as effective genus number (ENG) [27]. Calculations were performed for three power series: q = 0 (species richness), q = 1 (the exponent of the Shannon index), and q = 2 (the inverse of the Simpson index). Beta diversity was assessed using a distance matrix between samples, with the Jaccard index for binary data (presence/absence) and the Bray–Curtis measure for abundance data. Common and unique bacterial genera in the studied soils were visualized using InteractiVenn http://www.interactivenn.net [87]. The assignment of bacterial metabolic functions was based on their taxonomic classification, using the FAPROTAX database [88,89] in MACADAM software [28]. The data were presented using the SRplot online platform (bioinformatics.com.cn) [90].

## 5. Conclusions

Cadmium contamination significantly affected the structure, diversity, and functioning of the soil bacterial microbiome, resulting in a decrease in the abundance of cultivable organotrophic bacteria and actinobacteria, as well as the selection of bacteria tolerant to environmental stress. Although humic acids increased the abundance of organotrophic bacteria and actinobacteria, compost was most effective at mitigating the negative effects of Cd by stabilizing the structure of the bacterial community, supporting bacterial growth and improving functional stability of the microbial community. Among uncultivable bacteria, cadmium decreased the abundance of *Actinomycetota* and increased the abundance of *Pseudomonadota* and *Bacteroidota*, indicating a shift toward fast-growing (r-strategist) taxa. The application of organic amendments to cadmium-contaminated soil increased the abundance of *Pseudomonadota* and *Actinomycetota* (humic acids), *Pseudomonadota* (fermented bark), and *Bacillota* (compost). These results demonstrate that soil enriched with organic matter, regardless of type, can mitigate cadmium-induced damage to bacterial communities. The study demonstrated that both cadmium contamination and organic amendments significantly modified bacterial alpha and beta diversity. The magnitude and direction of these changes depended on the type of organic amendment applied. Bacterial alpha diversity was differentially affected by cadmium and organic amendments, with compost showing the most stabilizing effect on diversity indices. Beta diversity was influenced by both cadmium contamination and organic amendments, with fermented bark showing the strongest divergence in community composition. Cadmium altered the composition of the soil bacterial microbiome, whereas several bacterial genera belonging to the core microbiome, including *Sphingomonas*, *Nocardioides*, *Phenylobacterium*, and *Mucilaginibacter*, remained present across contaminated treatments, suggesting an important role in maintaining ecosystem stability. Our findings demonstrate that cadmium disrupts the structural and metabolic functions of bacterial communities. Organic matter added to the soil can partially mitigate the adverse effects of cadmium, although its effectiveness depends on the type of amendment used. Under the conditions of the experiment, the most favorable results were obtained when compost was applied at a dose of 3 g C kg^−1^ of soil, confirming its high potential for the remediation of contaminated soils. Therefore, it is necessary to develop more effective strategies to mitigate the degradation of soil microbial properties caused by excessive cadmium deposition and to extend research to include mycobiome responses and the direct assessment of microbial ecological and metabolic functions.

## Data Availability

The original contributions presented in this study are included in the article/Supplementary Materials. Further inquiries can be directed to the corresponding author.

## Supplementary Materials

The following supporting information can be downloaded at: https://www.mdpi.com/article/10.3390/ijms27135783/s1.

## Abbreviations

The following abbreviations are used in this manuscript:

C	uncontaminated soil
Cp	uncontaminated soil with compost
B	uncontaminated soil with fermented bark
H	uncontaminated soil with HumiAgra
Cd	soil contaminated with Cd^2+^
CdCp	soil contaminated with Cd^2+^ and compost
CdB	soil contaminated with Cd^2+^ and fermented bark
CdH	soil contaminated with Cd^2+^ and HumiAgra
