# Supplementary material for: Changes in Soil Bacteriobiome in Response to Organic Amendments and Cd2+ Stress
Source: Int J Mol Sci. 2026 Jun 26;27(13):5783. doi: 10.3390/ijms27135783 (PMC13361941; doi:10.3390/ijms27135783)
Supplement: Supplementary file 1 [file ijms-27-05783-s001.zip › ijms-4363590-supplementary.pdf]

International Journal of *Molecular Sciences*

MDPI

Supplementary Materials

Changes in Soil Bacteriobiome in Response to Organic Amendments and Cd^{2+} Stress

Agata Borowik, Jadwiga Wyszkowska*, Magdalena Zaborowska and Jan Kucharski

Table S1. Alpha diversity indices expressed as Hill numbers (Effective Number of Genus, ENG) across treatment soil. The table presents genus richness (q0), Shannon diversity (q1), and Simpson diversity (q2).

Objects	Diversity	Observed Sobs	Estimator	s.e.	Confidence Interval	
					LCL	UCL
B	Richness	252.00	252.00	0.59	252.00	253.16
B	Shannon	42.68	42.74	0.26	42.23	43.26
B	Simpson	16.60	16.61	0.12	16.38	16.84
C	Richness	212.00	212.25	3.13	212.00	218.38
C	Shannon	29.87	29.91	0.18	29.55	30.27
C	Simpson	10.59	10.60	0.08	10.44	10.75
Cd	Richness	272.00	272.50	2.23	272.00	276.87
Cd	Shannon	54.77	54.85	0.31	54.24	55.47
Cd	Simpson	23.56	23.57	0.18	23.22	23.92
CdB	Richness	206.00	206.00	0.52	206.00	207.02
CdB	Shannon	37.54	37.59	0.21	37.17	38.00
CdB	Simpson	18.11	18.11	0.11	17.89	18.33
CdCp	Richness	224.00	224.50	1.70	224.00	227.83
CdCp	Shannon	51.17	51.25	0.26	50.74	51.75
CdCp	Simpson	27.90	27.91	0.16	27.60	28.22
CdH	Richness	212.00	212.00	1.65	212.00	215.23
CdH	Shannon	31.83	31.87	0.17	31.54	32.19
CdH	Simpson	15.16	15.16	0.08	15.00	15.33
Cp	Richness	225.00	225.00	0.90	225.00	226.77
Cp	Shannon	50.86	50.94	0.24	50.47	51.40
Cp	Simpson	26.67	26.68	0.13	26.42	26.94
H	Richness	306.00	306.00	0.90	306.00	307.75
H	Shannon	54.88	54.97	0.32	54.34	55.60
H	Simpson	18.00	18.01	0.12	17.77	18.25

* Observed (Sobs) – number of observed taxa; Estimator – estimated total taxonomic richness; s.e. – standard error; LCL, UCL – lower and upper 95% confidence limits, respectively.

 https://doi.org/10.3390/ijms27135783

Table S2. The taxonomic classification of bacterial genera responsible for specific ecological and metabolic functions was determined using the MACADAM database.

Functional information	Genus
aerobic anoxygenic phototrophy	*Sphingomonas*
aerobic chemoheterotrophy	*Acidothermus, Blastococcus, Terrabacter, Asticcacaulis, Mesorhizobium, Bacillus, Rhodanobacter, Streptomyces, Marmoricola, Sphingobium, Cellvibrio, Nocardioides, Limnobacter, Dyella, Sphingomonas, Pedobacter, Paenibacillus, Phenylobacterium, Thermomonas, Hephaestia, Conexibacter, Mucilaginibacter, Phycicoccus, Gemmatimonas, Cellulosimicrobium, Pseudomonas, Knoellia, Lapillicoccus, Bryobacter*
animal parasites or symbionts	*Ramlibacter, Bacillus, Limnobacter, Pseudomonas, Paenibacillus, Enterobacter*
aromatic compound degradation	*Bacillus, Nocardioides, Pseudomonas, Sphingomonas, Sphingobium, Paenibacillus*
aromatic hydrocarbon degradation	*Pseudomonas, Sphingobium, Paenibacillus*
arsenate respiration	*Bacillus*
cellulolysis	*Bacillus, Acidothermus, Cellvibrio, Cellulosimicrobium, Pseudomonas, Streptomyces, Paenibacillus, Chitinophaga*
chemoheterotrophy	*Acidothermus, Thermoactinomyces, Enterobacter, Blastococcus, Terrabacter, Asticcacaulis, Mesorhizobium, Bacillus, Rhodanobacter, Streptomyces, Marmoricola, Lysobacter, Sphingobium, Cellvibrio, Nocardioides, Limnobacter, Dyella, Sphingomonas, Pedobacter, Paenibacillus, Phenylobacterium, Thermomonas, Chitinophaga, Conexibacter, Hephaestia, Mucilaginibacter, Phycicoccus, Gemmatimonas, Cellulosimicrobium, Pseudomonas, Knoellia, Lapillicoccus, Bryobacter*
chitinolysis	*Bacillus, Streptomyces, Lysobacter, Paenibacillus, Chitinophaga*
dark oxidation of sulfur compounds	*Bacillus, Limnobacter, Dyella, Pseudomonas, Rhodanobacter*
dark sulfite oxidation	*Dyella*
dark sulfur oxidation	*Dyella*
dark thiosulfate oxidation	*Bacillus, Limnobacter, Dyella, Pseudomonas, Rhodanobacter*
denitrification	*Pseudomonas, Bacillus, Nocardioides*
dissimilatory arsenate reduction	*Bacillus*
fermentation	*Bacillus, Cellulosimicrobium, Streptomyces, Paenibacillus, Enterobacter*
fumarate respiration	*Bacillus, Paenibacillus*
human gut	*Enterobacter*
human pathogens all	*Ramlibacter, Bacillus, Limnobacter, Pseudomonas, Enterobacter*
human pathogens pneumonia	*Ramlibacter, Bacillus, Pseudomonas, Limnobacter*
human pathogens septicemia	*Pseudomonas*
hydrocarbon degradation	*Pseudomonas, Streptomyces, Sphingobium, Paenibacillus*
invertebrate parasites	*Bacillus, Pseudomonas, Paenibacillus*
iron respiration	*Bacillus*
ligninolysis	*Bacillus, Pseudomonas, Sphingomonas, Streptomyces, Enterobacter*
mammal gut	*Enterobacter*
manganese oxidation	*Bacillus, Pseudomonas, Planifilum, Thermoactinomyces, Paenibacillus*
manganese respiration	*Bacillus*

https://doi.org/10.3390/ijms27135783

methanol oxidation	*Bacillus, Pseudomonas*
methylotrophy	*Bacillus, Pseudomonas*
nitrate ammonification	*Bacillus, Enterobacter*
nitrate denitrification	*Pseudomonas, Bacillus, Nocardioides*
nitrate reduction	*Conexibacter, Bacillus, Phycicoccus, Nocardioides, Dyella, Pseudomonas, Knoellia, Paenibacillus, Pseudarthrobacter, Cellulosimicrobium, Rhodanobacter, Streptomyces, Enterobacter, Terrabacter, Chitinophaga*
nitrate respiration	*Bacillus, Nocardioides, Pseudomonas, Paenibacillus, Streptomyces, Enterobacter*
nitrite ammonification	*Bacillus, Enterobacter*
nitrite denitrification	*Pseudomonas, Bacillus, Nocardioides*
nitrite respiration	*Bacillus, Nocardioides, Pseudomonas, Paenibacillus, Enterobacter*
nitrogen fixation	*Cellvibrio, Paenibacillus, Mesorhizobium, Sphingomonas*
nitrogen respiration	*Bacillus, Nocardioides, Pseudomonas, Paenibacillus, Streptomyces, Enterobacter*
nitrous oxide denitrification	*Pseudomonas, Bacillus, Nocardioides*
oil bioremediation	*Pseudomonas, Streptomyces*
photoheterotrophy	*Sphingomonas*
phototrophy	*Sphingomonas*
plant pathogen	*Bacillus, Pseudomonas, Paenibacillus, Sphingomonas, Streptomyces, Enterobacter*
plastic degradation	*Pseudomonas, Sphingobium, Sphingomonas*
respiration of sulfur compounds	*Bacillus*
thiosulfate respiration	*Bacillus*
ureolysis	*Bacillus, Phycicoccus, Dyella, Pseudomonas, Cellulosimicrobium, Pseudarthrobacter, Pedobacter, Streptomyces, Lysobacter, Paenibacillus, Terrabacter, Thermomonas, Mesorhizobium, Chitinophaga*
xylanolysis	*Bacillus, Nocardioides, Pseudomonas, Streptomyces, Thermoactinomyces, Paenibacillus*

https://doi.org/10.3390/ijms27135783

Table S3. A phenotypic profile of bacteria obtained from the IJSEM (International Journal of Systematic and Evolutionary Microbiology) database, determined using MACADAM with respect to taxonomic classification at the genus level.

Functional information	Genus
H_2S production	*Bacillus, Nocardioides, Dyella, Pseudomonas, Knoellia, Sphingomonas, Streptomyces, Marmoricola, Lysobacter, Paenibacillus*
acid phosphatase	*Luteimonas, Blastococcus, Terrabacter, Asticcacaulis, Bacillus, Rhodanobacter, Streptomyces, Marmoricola, Lysobacter, Sphingobium, Nocardioides, Dyella, Pseudarthrobacter, Sphingomonas, Pedobacter, Paenibacillus, Chitinophaga, Conexibacter, Mucilaginibacter, Phycicoccus, Cellulosimicrobium, Pseudomonas, Knoellia, Lapillicoccus, Bryobacter*
aesculin hydrolysis	*Luteimonas, Blastococcus, Terrabacter, Asticcacaulis, Mesorhizobium, Bacillus, Rhodanobacter, Streptomyces, Marmoricola, Lysobacter, Sphingobium, Nocardioides, Dyella, Pseudarthrobacter, Sphingomonas, Pedobacter, Planifilum, Paenibacillus, Luteibacter, Chitinophaga, Mucilaginibacter, Phycicoccus, Cellulosimicrobium, Pseudomonas, Knoellia, Lapillicoccus, Bryobacter*
alkaline phosphatase	*Luteimonas, Blastococcus, Asticcacaulis, Bacillus, Rhodanobacter, Streptomyces, Marmoricola, Lysobacter, Sphingobium, Nocardioides, Dyella, Pseudarthrobacter, Sphingomonas, Pedobacter, Paenibacillus, Phenylobacterium, Chitinophaga, Conexibacter, Mucilaginibacter, Phycicoccus, Cellulosimicrobium, Pseudomonas, Knoellia, Bryobacter*
alpha-galactosidase	*Conexibacter, Bacillus, Mucilaginibacter, Phycicoccus, Nocardioides, Luteimonas, Dyella, Sphingomonas, Rhodanobacter, Pedobacter, Streptomyces, Asticcacaulis, Planifilum, Sphingobium, Paenibacillus, Terrabacter, Chitinophaga*
catalase positive	*Luteimonas, Blastococcus, Terrabacter, Mesorhizobium, Bacillus, Rhodanobacter, Streptomyces, Lysobacter, Sphingobium, Nocardioides, Limnobacter, Dyella, Pseudarthrobacter, Sphingomonas, Pedobacter, Planifilum, Paenibacillus, Phenylobacterium, Luteibacter, Thermomonas, Chitinophaga, Conexibacter, Mucilaginibacter, Phycicoccus, Cellulosimicrobium, Pseudomonas, Knoellia, Lapillicoccus, Bryobacter*
gelatinase	*Luteimonas, Terrabacter, Bacillus, Rhodanobacter, Streptomyces, Marmoricola, Lysobacter, Sphingobium, Nocardioides, Dyella, Sphingomonas, Pedobacter, Planifilum, Paenibacillus, Luteibacter, Chitinophaga, Conexibacter, Mucilaginibacter, Phycicoccus, Pseudomonas, Knoellia, Bryobacter*
indole production	*Bacillus, Nocardioides, Pseudomonas, Pseudarthrobacter, Sphingobium, Paenibacillus, Mesorhizobium, Chitinophaga*
nitrate reduction to nitrite	*Luteimonas, Terrabacter, Asticcacaulis, Bacillus, Rhodanobacter, Streptomyces, Marmoricola, Lysobacter, Sphingobium, Nocardioides, Dyella, Pseudarthrobacter, Sphingomonas, Paenibacillus, Phenylobacterium, Chitinophaga, Conexibacter, Mucilaginibacter, Phycicoccus, Cellulosimicrobium, Pseudomonas, Knoellia*
oxidase positive	*Luteimonas, Blastococcus, Terrabacter, Mesorhizobium, Bacillus, Rhodanobacter, Streptomyces, Lysobacter, Sphingobium, Nocardioides, Limnobacter, Dyella, Sphingomonas, Pedobacter, Paenibacillus, Phenylobacterium, Luteibacter, Thermomonas, Chitinophaga, Mucilaginibacter, Phycicoccus, Pseudomonas*
pyrazinamidase	*Pseudarthrobacter*
urease positive	*Bacillus, Mucilaginibacter, Phycicoccus, Nocardioides, Luteimonas, Dyella, Pseudomonas, Limnobacter, Cellulosimicrobium, Pseudarthrobacter, Sphingomonas, Pedobacter, Streptomyces, Sphingobium, Paenibacillus, Terrabacter, Mesorhizobium, Chitinophaga*

https://doi.org/10.3390/ijms27135783
